# Clinical Relevance of State-of-the-Art Analysis of Surface Electromyography in Cerebral Palsy

**DOI:** 10.3389/fneur.2020.583296

**Published:** 2020-12-11

**Authors:** Germana Cappellini, Francesca Sylos-Labini, Carla Assenza, Laura Libernini, Daniela Morelli, Francesco Lacquaniti, Yury Ivanenko

**Affiliations:** ^1^Laboratory of Neuromotor Physiology, IRCCS Santa Lucia Foundation, Rome, Italy; ^2^Department of Pediatric Neurorehabilitation, IRCCS Santa Lucia Foundation, Rome, Italy; ^3^Department of Systems Medicine, Centre of Space Bio-medicine, University of Rome Tor Vergata, Rome, Italy

**Keywords:** cerebral palsy, abnormal development, muscle pathophysiology, surface electromyography, spinal locomotor output, rehabilitation, clinical application

## Abstract

Surface electromyography (sEMG) can be used to assess the integrity of the neuromuscular system and its impairment in neurological disorders. Here we will consider several issues related to the current clinical applications, difficulties and limited usage of sEMG for the assessment and rehabilitation of children with cerebral palsy. The uniqueness of this methodology is that it can determine hyperactivity or inactivity of selected muscles, which cannot be assessed by other methods. In addition, it can assist for intervention or muscle/tendon surgery acts, and it can evaluate integrated functioning of the nervous system based on multi-muscle sEMG recordings and assess motor pool activation. The latter aspect is especially important for understanding impairments of the mechanisms of neural controllers rather than malfunction of individual muscles. Although sEMG study is an important tool in both clinical research and neurorehabilitation, the results of a survey on the clinical relevance of sEMG in a typical department of pediatric rehabilitation highlighted its limited clinical usage. We believe that this is due to limited knowledge of the sEMG and its neuromuscular underpinnings by many physiotherapists, as a result of lack of emphasis on this important methodology in the courses taught in physical therapy schools. The lack of reference databases or benchmarking software for sEMG analysis may also contribute to the limited clinical usage. Despite the existence of educational and technical barriers to a widespread use of, sEMG does provide important tools for planning and assessment of rehabilitation treatments for children with cerebral palsy.

## Introduction

Cerebral palsy (CP) is the most common form of motor disability in childhood. It describes a group of permanent disorders of movement and posture, caused by disturbances in the fetal or infant brain (1). The clinical manifestations of CP vary greatly in the type of movement disorder and the degree of functional disability. It is often characterized by impaired coordination, muscle weakness, spasticity, hyperreflexia, hypertonia, clonus, spasms and co-contraction (2, 3). Children with CP have a variety of symptoms and CP is often accompanied by other disorders such as cognitive dysfunction, communication problems, deficits of vision, epilepsy, etc. (4, 5). Currently there are multiple symptomatic treatments being used, such as physical therapy (e.g., therapeutic exercises according to Bobath or Vojta; constraint induced therapy) and orthotics (e.g., foot, ankle-foot, knee-ankle-foot, hip-knee-ankle-foot orthoses), pharmacologic treatments (systemic medications: e.g., oral baclofen, diazepam, tizanidine, dantrolene, local intramuscular injection of botulinum toxin A), neurosurgical procedures (e.g., selective dorsal rhizotomy, deep brain stimulation), surgical neuro-orthopedic interventions that may include muscle tendon lengthening to correct retractions, interventions for re-establishing muscle balance through tendon transfer, rotational osteotomies for correcting bone deformities of the spine or lower limbs (6–12). Instrumentation-based assessment of functional disability is essential both for understanding the mechanisms of impaired movement control and for evaluation of treatment.

In recent decades, significant developments have occurred in many electrodiagnostic studies that provide powerful quantitative approaches to instrumentation-based assessments in cardiology (electrocardiology), neurology (electroencephalography), skeletal muscle functioning (surface electromyography, sEMG). In this work we will specifically focus on sEMG. It can be used for evaluation of the integrity of the nervous system in neurological disorders with motor deficit, playing an important role in neurorehabilitation and predicting the outcome of neuromuscular disorders (13–17). Multi-muscle sEMG recordings provide information on muscular recruitment/de-recruitment capability, fatigue, synergistic activation, co-contractions, as well as contribute to the evidence for the efficacy of the rehabilitation plan (18, 19). Quantitative sEMG can be used as a practical, relatively simple and non-invasive tool and a screening method adopted by medical doctors and physiotherapists. However, although sEMG study is an important tool in neurorehabilitation, the limited clinical use and almost no teaching in the physical therapy schools of most countries represent a contradiction (20).

This article specifically reports the potential clinical value of techniques based on sEMG in neurorehabilitation medicine of children with CP and addresses the barriers limiting a widespread clinical usage of sEMG for this patient population. In the first section, we will briefly consider the motor impairments that result from a lesion occurring in the developing brain and describe sEMG applications for the assessment of neurological impairments and for performing interventions/treatment in children with CP. In the second section, we will present the results of a survey directed to the physiotherapists, neuro-developmental disorders therapists and medical doctors of a department of pediatric rehabilitation related to the barriers limiting a widespread clinical use of sEMG techniques in clinical assessment and neurorehabilitation of children with CP.

## Motor Impairments and sEMG Applications in CP

### Motor Impairments

One of the most widespread and used classifications of CP is that proposed by Surveillance of Cerebral Palsy in Europe. According to their criteria, all CP subtypes have an abnormal pattern of movement and posture and classification is applied in a hierarchical manner using the predominant type of muscle tone and movement abnormality, resulting in the following categories: spastic, dyskinetic and ataxic (21). The most common type of CP, on terms of motor control, is spastic CP, which affects ~70–80% of the population of children with CP and results in impaired sensory-motor control, muscle weakness and muscle hyper-resistance (22). Objective functional scales have been employed to assess individuals with CP such as the Gross Motor Function Classification System (GMFCS) for assessing functional mobility and motor skills related to both lower and upper limbs. Parallel classification scales have been developed for assessing upper extremity function in CP, such as the Bimanual Fine Motor Function Scale (BFMF) and the Manual Ability Classification System (MACS) (22).

Comprehensive descriptions of motor impairments in children with CP have been reported in numerous studies both for lower and upper limbs. Children with CP may develop various motor dysfunctions, including dystonia, contractures, hyperreflexia, muscle weakness, lack of coordination (23, 24), increased passive musculotendinous stiffness (25), increased cocontraction of antagonists (26, 27), structural changes in muscle fibers and connective tissues (28–32).

**Figure 1:**
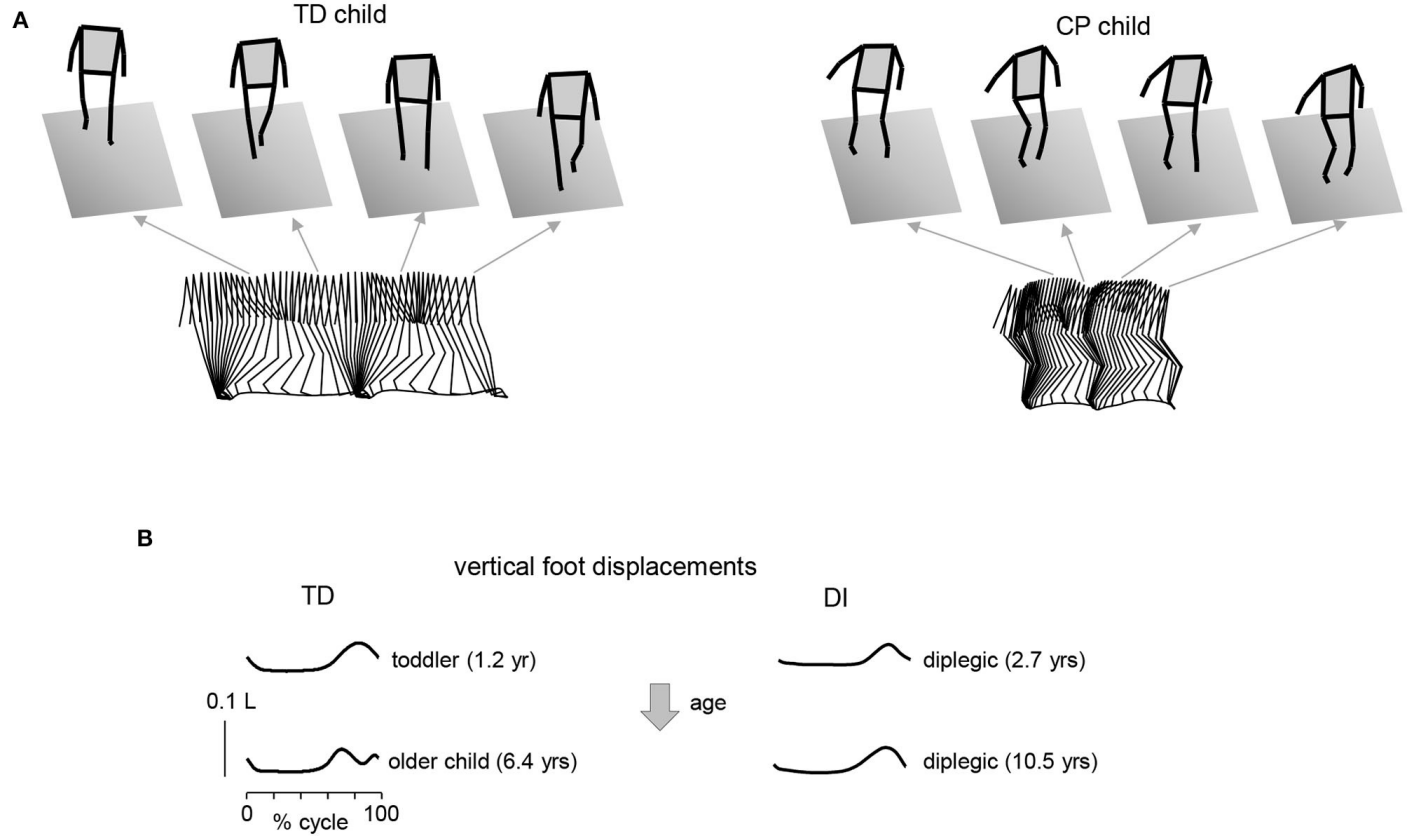
Gait impairments in children with cerebral palsy. **(A)** Stick diagrams (3D, sagittal on the bottom) of 2 consecutive strides in one TD child (5.7 yrs) and one diplegic child (6.3 yrs). Note typical two peaked profile of vertical hip position during one stride in TD child (pendulum mechanism of walking) and variable pattern in child with CP. **(B)** Vertical foot displacements averaged across 45–60 strides during overground walking at self-selected speed for two TD children (1.2 and 6.4 yrs) and two diplegic children (2.7 and 10.5 yrs). Vertical foot displacements are expressed in relative units (normalized by the limb length L). Patterns are plotted vs. the normalized gait cycle. Adapted from Cappellini et al. (37).

Gait impairments in CP are also typical (33). In particular, children with CP show difficulties in gait maturation and a lack of some major features of adult gait (pendulum mechanism of walking, foot trajectory control), frequent problem of foot drop associated with impaired ability to dorsiflex the ankle, difficulties in hip extension and ankle joint plantarflexion at end stance, excessive leg muscle co-activation, increased proprioceptive reflexes, and delayed or impaired maturation of the spinal pattern generation output (27, 34–38). Some characteristic features of gait are illustrated in Figure 1. In line with the general hypothesis of delayed maturation (39), many idiosyncratic features of gait in older children with CP resemble those in typically developing (TD) children at the onset of independent walking (37), for instance, the noticeable single-peak foot lift (Figure 1B) and a lack of stereotyped vertical trunk displacements resulting from the pendulum mechanism of walking. The adult two-peaked foot trajectory, representing an accurate endpoint control with a minimum at midswing (40–42), is usually not observed in children with CP; instead, a single peak of the foot lift, typical for TD toddlers, can be frequently seen across all sampled ages in CP (37) (Figure 1B).

Since any reflection on functional disability in CP should consider the mechanisms and methods of their assessment, sEMG monitoring may be useful for assessing and treatment of motor impairments and various examples will be considered below.

### sEMG Applications

#### Background

Since the discovery of sEMG in 1912, myoelectric activity measurements provided many examples of normal and pathological skeletal muscle function, improved our knowledge about the neural control of movement and contributed to the development of clinical applications (43). sEMG registers the electrical potential at the surface of the skin associated with the summation of multiple action potentials of individual muscle fibers during their contraction and thus provides a direct measure of muscle contraction/relaxation activity controlled by the nervous system. One can record muscle activity by placing one or more pairs of electrodes on the skin over a muscle and sEMG can be used to estimate the global net firing of spinal motoneurons (MN) innervating that muscle, since it increases fairly linearly with the sum of rectified motor unit action-potentials at least over the physiological range of 0–50% contraction levels (44–46). The amplitude and spectral characteristics of the sEMG signal depend on the anatomical and physiological properties of muscles and subcutaneous tissue thickness. Surface EMG-derived indices have an important role as outcome measures to evaluate the responsiveness to treatments (47, 48). In some cases, the visual inspection of sEMG traces is easy to perform and data are easy to interpret, as in the case of a complete lack of muscle activity (paresis). In many clinical applications (e.g., support to the surgical planning) there is no need for algorithms and raw data may support the clinical decision making [e.g., (49, 50)]. However, some performance indicators, factors and processing methods, such as the determination of the onset/offset of sEMG bursts or amplitude normalization (43), require attention and agreement between the users, and in particular, the interpretation of sEMG signals with respect to muscular coordination requires some caution (51). For this reason, to support the interpretation of sEMG signals, different signal processing methodologies were developed (52).

Standard measurements and processing procedures of the sEMG signals are in great demand for a better understanding of neuromuscular control and are important for various biomedical applications and clinical diagnosis. In clinical practice, real-time sEMG can be used by physiotherapists as control if the movement requested is performed by the proper target muscle or by means of compensatory mechanisms, as a direct measurement of variations consequent to mobilization, verticalization, trunk fixation, or to assess the effect of different orthoses on muscle activation, which can vary toward or away from the normal pattern. Below we consider examples of using sEMG in children with CP for assessing neurological impairments and performing interventions.

#### Assessing Muscle Activity and Motor Dysfunctions

Walking is typically considered one of the most essential activities of daily living (53, 54). Clinical gait analysis is therefore useful and can get insights into the complexity and deficits in the control of pathological gait, and be integrated into the clinical decision-making of individuals with gait disorders (55). Motor problems in children with CP can be associated with excess symptoms such as hypertonia, spasticity, spasms, hyperreflexia, and deficiency symptoms such as muscular weakness, apraxia, ataxia, loss of selective activation of muscles (56). The latter feature is an important determinant of motor control in children with CP and can be used to monitor gross motor function progress over time (57). sEMG recordings may provide a quantitative assessment of coactivation and the degree of selective activation of muscles rather than using subjective estimates of muscle coordination with a low sensitivity (58). Moreover, sEMG is suitable for the detection of coactivation of agonist and antagonist muscles, so that physiological activation patters could be distinguished from pathological ones. Management and rehabilitation processes of children with CP can be improved using electromyographic techniques (59). Particularly, sEMG analysis in children with CP can also be used for surgical planning (60).

It is worth noting that the muscles are often weak and atrophic in children with CP, resulting in significantly reduced volumes in leg muscles and in bone changes (28, 29, 31, 61, 62). Therefore, interventions increasing muscle length or strength are beneficial. For instance, sEMG can be used to monitor and accomplish targeted muscle contraction in children with CP in order to prescribe exercise programmes for muscle strengthening and their effectiveness (63).

**Figure 2:**
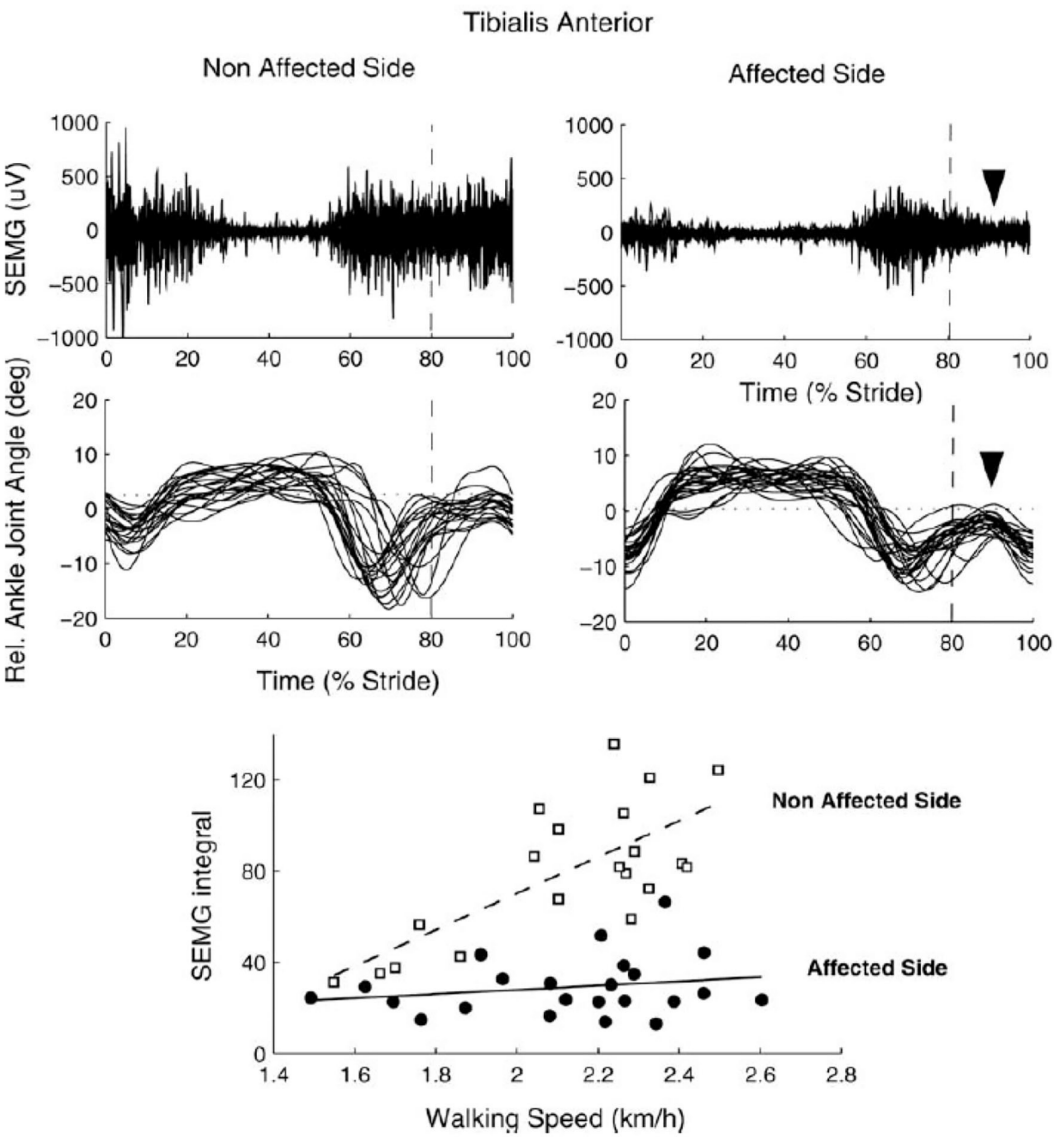
sEMG of tibialis anterior muscle during walking in one 8 yrs child with congenital hemiparesis. Upper panels: examples of sEMG plotted vs. the normalized gait cycle. Middle panels: ankle joint angles of individual strides. Lower panels: speed-dependent recruitment of TA (quantified as sEMG integral or the mean amplitude of rectified sEMG over the period) on the affected and non (least) affected side. Note reduced paretic component of TA around foot contact at end swing along with reduced foot dorsiflexion (upper and middle panels) and insufficient up-scaling of TA activity with speed (lower panel) on the affected side [reproduced from Frigo and Crenna (64) with permission].

**Figure 3:**
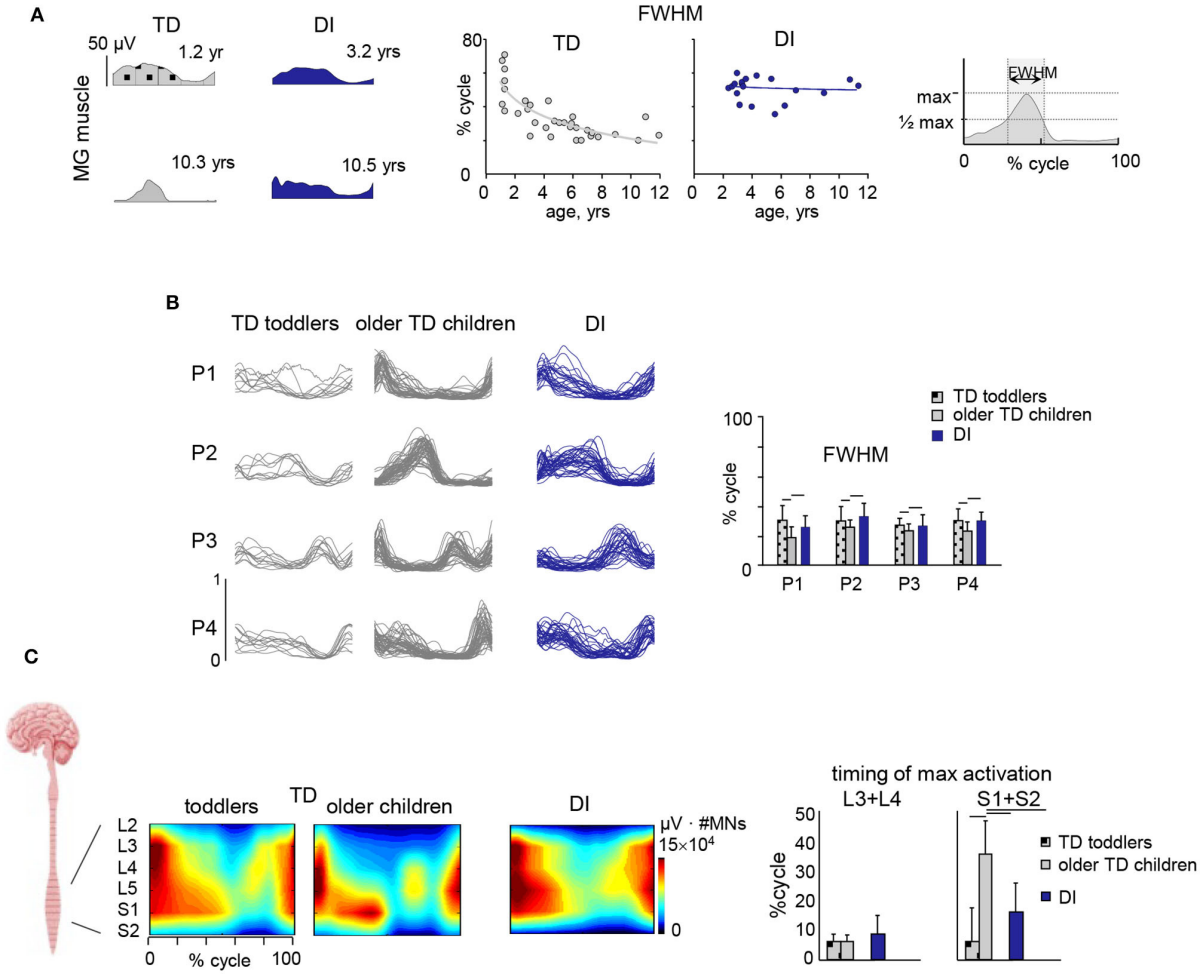
Muscle activity locomotor output and its impairments in children with CP. **(A)** Developmental trend for the duration of muscle (medial gastrocnemius, MG) activity. From left to right: examples of MG activity in two TD children (1.2 and 10.3 yrs) and two diplegic children (3.2 and 10.5 yrs), and duration of MG activity (full width at half maximum, FWHM, see right panel) as a function of age (continuous lines represent exponential fittings). Note significantly wider sEMG activity in CP, independent of age. **(B)** Statistical analysis of sEMG patterns: basic activation patterns P1–P4 consistent across individual children. Right panel – mean (+SD) FWHM of P1–P4. TD toddlers aged 1–1.2 yrs, older TD children aged 2.1–11.8 yrs and DI children aged 2.3–11.1 yrs. Modules were ranked based on their best similarities. Note significantly wider patterns in CP. **(C)** Segmental motoneuronal (MN) output in TD toddlers, TD older children and diplegic children estimated by mapping sEMG activity patterns of 11 simultaneously recorded lower limb muscles onto the approximate rostrocaudal location of the motor pools of the corresponding muscles (averaged across children and normalized to the mean number of MNs in spinal segments L2–S2) and plotted as a function of gait cycle. Output pattern for each segment L2–S2 was reconstructed by averaging all rectified sEMGs corresponding to that segment [for details, see (66)] and plotted in a color scale. To visualize a continuous smoothed rostrocaudal spatiotemporal activation of the spinal cord, we used a filled contour plot. To account for size differences in MN pools at each spinal level, this segmental activity value (in μV) was then multiplied by the segment-specific number of MNs, taken from Tomlinson and Irving (67). Right panel shows timing (+SD) of maximum activation of sacral (S1 + S2) and lumbar (L3 + L4) segments. Significant differences with respect to older TD children are indicated by lines over bars. Adapted from Cappellini et al. (37).

sEMG is commonly used to assess muscular coordination in clinical gait analysis but could also be used in functional diagnosis or in the monitoring of therapeutic outcomes. In particular sEMG could be useful for the assessment of the “paretic component,” i.e., defective activation of peripheral muscle effectors on most affected side of hemiplegic children. Reduced and insufficient speed dependent modulation of the ankle dorsiflexors' activity (e.g., tibialis anterior, TA) around foot contact at end swing (and to a lesser degree at end stance) can significantly affect the ankle dorsiflexion torque and consequently the foot trajectory in children with CP (Figure 2) (64). The TA activity frequently demonstrates only one major peak at lift-off at the onset of swing (on the most affected side of hemiplegic and on both sides in diplegic children) with respect to two prominent peaks in TD children [Figure 2, see also (37)]. This TA pattern is likely associated with impaired foot trajectory control. Other intrinsic and extrinsic foot muscles contribute to flexion/extension of the ankle and metatarsophalangeal joints as well (65) and their impaired activity might also limit ankle dorsiflexion and foot varus deviation in children with CP. However, a registration of sEMG activity of intrinsic foot muscles is challenging (e.g., due to crosstalk) and was not systematically performed for clinical gait assessments. Furthermore, there is a lack of important age-related changes of sEMG characteristics in children with CP. For instance, in TD children, there is a progressive reduction of sEMG burst durations with age and corresponding spatiotemporal characteristics of the spinal motor pool output, likely reflecting an essential developmental aspect of muscular control optimization (37). In children with CP, these characteristics of motoneron output are similar to those at the early stages of development in TD children (Figure 3A).

Functional corticospinal connectivity in CP can be assessed by estimating the oscillatory drive of the motor cortex to the spinal cord using coherence analysis of sEMG signals within and between muscles. Indeed, in children with CP, there is a frequent problem of foot drop associated with impaired control of the ankle dorsiflexors (Figure 2) and reflected also in reduced TA sEMG-sEMG coherence in the beta and gamma frequency bands associated with impaired functional corticospinal connectivity (68). Such sEMG-sEMG coherence assessment can be used for monitoring of therapeutic outcomes. For instance, 4 weeks of intensive training of walking on the inclined surface can reduced foot drop and significantly improve the ankle joint control in children with CP along with improved functional corticospinal connectivity and increased beta and gamma oscillatory drive to motoneurons (69).

#### Hyperreflexia

Hyperreflexia is a frequent feature in neurological disorders characterized by a velocity-dependent increase in tonic stretch reflexes (muscle tone) with exaggerated tendon jerks, resulting from hyper-excitability of the stretch reflex (70). sEMG examination is indispensable for detecting the presence, contribution and interference of the spastic component with walking pattern, augmenting motor unit recruitment, enhancing stretch responses during walking and coactivating muscles during specific phases or through the whole gait cycle (23, 71). Accordingly, hyperreflexia can significantly influence the locomotor movements, assist joint/segment stability or impede movements during muscle lengthening, limit ranges of angular motion, and may necessitate extra efforts or reorganization of muscle responses to compensate for these abnormalities. There is growing consensus that it is important to distinguish different contributions to joint hyper-resistance, i.e., non-neural originating from passive tissue properties, and neural originating from background muscle activity and stretch hyperreflexia. sEMG provides a mean to identify the contribution of muscle activity to muscle hyper-resistance (72).

Clinical analysis of muscle activity is thus necessary for deciding whether to intervene or not and in particular for determining the degree of post-treatment reduction of the spastic component. Experiments and data analysis using muscle models confirmed a tight coupling between kinematics and motor output in children with spastic diplegia, for instance, during the phases of lower limb muscle lengthening in the gait cycle (73). In particular, atypical stretch responses were more easily produced around the time of foot ground contact during lengthening contractions than at other moments of the gait cycle. The above findings point toward an essential role of sEMG measurements in the clinical evaluation, understanding of spastic muscle dysfunction in children with CP and improving the outcomes of neurorehabilitation (71).

#### Muscle Fatigue

Children with CP might have higher levels of activation in specific muscles and/or large amounts of coactivation of agonist and antagonist muscles in the same joint, which could increase muscle fatigue. Mechanical manifestations of muscle fatigue are defined as a reduction in the force-generating capacity of the neuromuscular system, which occurs during sustained activity (74). Muscle fatigue is usually divided into peripheral and central fatigue (75). Peripheral mechanical fatigue is generally a loss of force-generating capacity due to processes distal to the neuromuscular junction, whereas central is described as progressive reduction in voluntary activation. sEMG could be used to assess these changes in neuromuscular activation associated with peripheral fatigue.

Typically a decrease in frequency and an increase in root mean square of sEMG signals are interpreted as myoelectric manifestations of muscle fatigue (76, 77). Fatigue itself is not a physical variable. Its evaluation requires the definition of indices based on physical variables that can be measured, such as, for example, force or torque, power, angular velocity of a joint, or variables associated with the single motor unit, such as the conduction velocity, or with the sEMG signal, such as amplitude, spectral mean or median frequency. The conduction velocity is the main index, it decreases more or less rapidly depending on the level of contraction and is generally measured during isometric constant force contraction. The measurement of muscle fatigue in CP using sEMG reveals differences relative to the age-matched controls (78, 79).

#### Neuromuscular Electrical Stimulation

Neuromuscular or functional electrical stimulation (FES) is an example of the appropriate usage of temporal characteristics of sEMG recordings for determining the application of multi-channel electrical stimuli to superficial skeletal muscles in order to control, compensate and/or correct their contractions (80–85). In addition to a physiotherapy programme, it emphasizes task specificity, motor learning, and positive effects (86). It provides input while the child is engaged in a motivating, goal-directed activity. The decision about which muscles to stimulate is based on the biomechanics and required sEMG patterns during effective performance of the action. The child is an active participant and is encouraged to initiate movement. A single-case study reports that a boy aged 6.7 years learned to perform a number of tasks, including tying his shoelaces, after 24 sessions of stimulation to wrist extensors, finger flexors and extensors, with resisted exercises and task training (87). A period of wearing a dorsal wrist splint made of orthoplast was included to help him with his task practice. This study shows that such intervention is feasible with children and can be remedial. More recent studies of functional electrical stimulation applied to wrist extensor muscles found improvements in hand use (88–90). Other studies of children with diplegia found an increase in walking speed and muscle strength (80, 91, 92).

#### Electromyographic Biofeedback

Some studies used electromyographic biofeedback as a non-invasive, safe, and effective treatment for children with CP (93–95). Children with bilateral and unilateral CP may benefit from sEMG biofeedback therapy for various tasks including both upper and lower extremities. sEMG biofeedback treatment is an active rehabilitation training capable of detecting the signals of muscle contraction through auditory and/or visual feedback for motivating child's involvement and stimulating the recovery or improvement of the limb control (96). Some previous studies demonstrated the positive clinical effects of using sEMG biofeedback in improving upper limb dysfunction in persons with CP (94, 97, 98), the motor outcomes at the ankle joint, the strength of muscle contractions, range of motion, and walking speed (93, 99, 100).

#### Characterization of Multi-Muscle Activity Regularities (Basic Muscle Modules)

To perform a movement, the central nervous system (CNS) should engage many muscles and control corresponding forces exerted around joints involved. Furthermore, muscles differ in the fiber composition (slow, fast) and structure (pinnate, parallel fibers, etc.), may be divided into compartments and comprise quite different number (thousands) of motor units. During locomotion or other movements, tens of muscles are active and need to be coordinated simultaneously. The idea that the CNS can control the complexity of interactions to promote a certain motor act by adopting a modular decomposition and therefore a limited number of primitives has recently received a lot of attention (101). In the last years researchers showed evidence that muscle activation patterns, represented by the sEMG envelopes of a few muscles, can be decomposed into a limited number of “basic” functions or patterns, called “synergies” or “primitives” (37, 102–104). These primitive patterns can be combined, with different individual weights, and produce the corresponding compound task-related muscle activations to perform the movement. It has been hypothesized that the CNS simplifies muscle control through modularity, using these basic synergies (primitives) to activate muscles in groups (105).

This approach has had a large impact on the analysis of motor control in the field of neurorehabilitation since it implies that the CNS generates forces and movements by optimizing the control strategy of either individual muscles or (more likely) muscle synergies (106). Concerning application of this approach to neurorehabilitation of children with CP, several studies evaluated impairments in the modular organization of multi-muscle activity patterns and alterations of muscle synergies (37, 100, 107–120). The multi-muscle activity analysis through non-negative matrix factorization revealed that sEMG activity regularities and patterns can be adequately captured and represented by a small number of temporal components during walking in children with CP and in TD children (Figure 3B). Such analysis showed a comparable spatiotemporal organization of the motor output in both groups, but noticeably wider temporal basic activation patterns in CP, similar to the patterns of younger TD toddlers (Figures 3A,B) (37). Reduction of dimensionality (fewer muscle synergies) reported in some studies [e.g., (107, 109, 110)] may depend on the relatively small number of analyzed muscles (121–123) and/or the method used to define the minimum number of modules (37, 124, 125). Moreover, the observed phenomenon of widening seems to be a characteristic feature of CP gait and does not depend on the number of modules used in the sEMG decomposition procedure (126).

#### Spinal Segmental Motoneuron Output

The final neural output of spinal locomotor circuitry is represented by the spatiotemporal activation of α-motoneurons (MN). It can be evaluated indirectly by using sEMG recordings from a large number of lower limb muscles and mapping their activity patterns onto the approximate rostrocaudal location of the motor pools of the corresponding muscles in the lumbosacral enlargement (66, 127, 128). The implicit assumption is that the rectified sEMG provides an indirect estimate of the net firing of spinal MNs innervating that muscle (44–46, 127). In essence, to reconstruct the motor-pool output pattern of any given spinal segment innervating limb muscles, all rectified sEMG-waveforms corresponding to that segment are averaged using appropriate weighting coefficients (66, 127). In general, each muscle is innervated by several spinal segments, and each segment supplies several muscles (129), so that one may estimate the segmental MN output by adding up the contribution of each muscle to the total activity in each spinal segment according to the published myotomal charts of segmental innervation in humans. The analysis of motor pool activation using multi-muscle sEMG can also be complemented by a statistical analysis of the muscle activity profiles and their decomposition into a small set of so-called muscle modules or common basic activation components (see the previous section) as a means to look backward from the periphery to the spinal cord motor programming and output (130, 131). There are now several studies that evaluated the spinal locomotor output, its spatiotemporal organization and impairment in children with CP (37, 100, 107–120).

Figure 3C illustrates the spinal maps of MN activation during walking and typical features of motor output impairment in children with CP compared to TD children obtained using the averaged rectified sEMG profiles of multiple leg muscles as an indirect measure of the net MN firing in the spinal cord. TD children show a notable functional reorganization and maturation of the MN output with increasing age, consisting in more narrow loci of MN activity and a progressive shift of the timing of maximum activation of sacral segments toward later stance (Figure 3C). By contrast, this developmental trend in children with CP is lacking. They show very limited reduction in the muscle activity pattern durations with age and limited changes in the timing of lumbar and sacral motor pool activation over the gait cycle (37), in line with the idea that early injuries to developing brain substantially affect the maturation and functioning of the spinal pattern generation circuitry.

## Results of a Survey on Clinical Relevance of sEMG in a Department of Pediatric Rehabilitation

A number of “barriers” exist limiting the widespread application of sEMG techniques in clinical assessment and neurorehabilitation of children with CP. We aimed at examining these barriers by gathering information from the clinicians involved in pediatric rehabilitation to generate opinion on the current use of sEMG and its clinical utility. We think that getting some background views and perspectives will help our understanding of the current applications of sEMG in neurorehabilitation of children with CP as well as of the potential obstacles to its use in the clinical setting in this particular area. To this end, we conducted a survey directed to the physiotherapists, neuro-developmental disorders therapists and medical doctors related to the barriers limiting a use of sEMG techniques in clinical assessment and neurorehabilitation of children with CP.

### Participants

**Figure 4:**
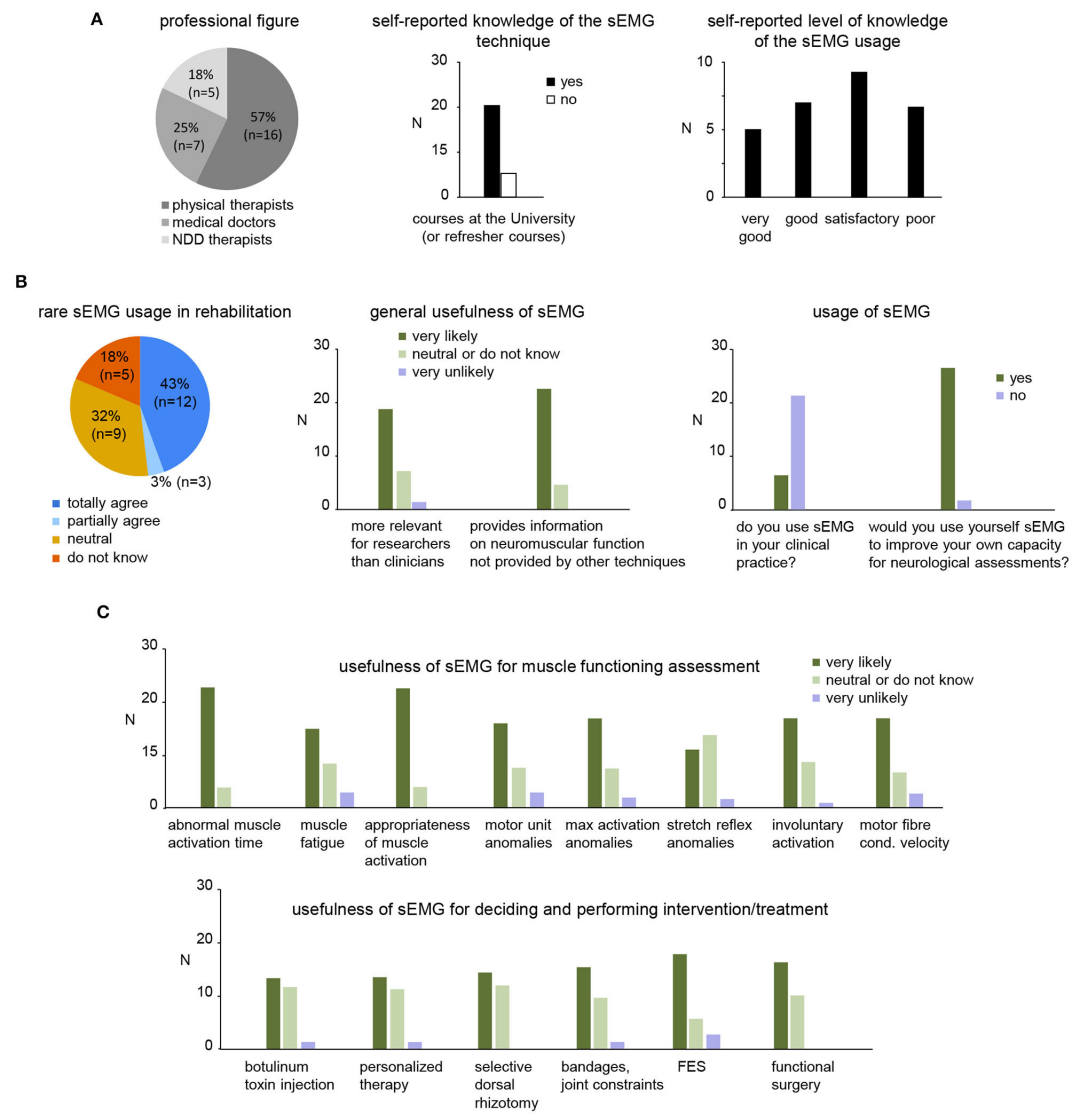
Results of survey to address the usefulness of sEMG in clinical practice for children with CP. **(A)** Pie chart showing the number and percentage of each group of participants that completed the survey (twenty-eight returned completely survey questionnaires: 7 medical doctors, 5 neuro-developmental disorders (NDD) therapists and 16 physiotherapists) and self-reported knowledge of the sEMG techniques and usage. **(B)** General assessment of the sEMG usage in rehabilitation. Left panel: pie chart showing the percentage of the participants that agree or disagree of rare sEMG use in clinical neurorehabilitation. Middle panel: general relevance of sEMG for research and clinical usage. Right panels: usage of sEMG and willing to use by participants of the survey. **(C)** Usefulness of sEMG for functional assessment (upper panel) and performing/defining an intervention (lower panel).

An online survey involving the personnel of the Department of Pediatric Neurorehabilitation of the IRCCS Santa Lucia Foundation was conducted. Of the 36 invitations sent, 28 invitees completed survey questionnaires. Professional background was varied, with 16 (57%) physical therapists, 7 (25%) medical doctors, and 5 (18%) neuro-developmental disorders therapists (Figure 4A, left panel).

### Survey Questionnaire

We have prepared a 34-item (Table 1) online survey. To address the barriers to clinical use of sEMG, we included in the survey questions about the potential added value that could be provided by sEMG-based assessments but also the reasons of the minimal use in the clinical practice. The questionnaire also included information about participants: self-reported knowledge about the sEMG techniques obtained during studies at the university and/or refresher courses, self-reported level of knowledge of the sEMG usage, and self-reported usage of sEMG in their own clinical practice. Participants were invited to participate via an e-mail and an online survey (using *Google Forms*) was used to collect the answers electronically. They were requested to respond to the questionnaire by selecting one of the answers to each statement (Table 1).

**Table 1:** Survey items of the questionnaire to address the usefulness of sEMG in clinical practice for children with CP.

**Statement**	**Possible answers (one answer for each statement)**
Self-reported knowledge of the sEMG technique: did you address this topic during your studies at the university and/or refresher courses?	yes no
Self-reported level of knowledge of the sEMG usage	very good good satisfactory poor
sEMG is rarely used in clinical neurorehabilitation	totally agree partially agree neutral do not know
General usefullness of sEMG in the field of neurorehabilitation 1. sEMG is currently more relevant for researchers than clinicians 2. sEMG provides information on neuromuscular function not provided by other techniques	very likely neutral/do not know very unlikely
Usage of sEMG and willing to use by participants of survey 1. do you use sEMG in your clinical practice? 2. would you use yourself sEMG to improve your own capacity for neurological assessments?	yes no
Usefulness of sEMG for muscle functioning assessment. sEMG can be useful to: 1. outline the abnormal timing of muscular actions during movements (i.e., gait, motor tasks) 2. evaluate muscle fatigue 3. evaluate the appropriateness of muscle activation in specific motor acts 4. identify pathological patterns of motor unit behavior 5. evaluate anomalies of maximal voluntary activation 6. characterize the stretch reflex 7. characterize involuntary muscle activations (e.g., dystonia, ataxia, spasticity) 8. characterize the motor fibres' conduction velocity	very likely neutral/do not know very unlikely
Usefulness of sEMG for deciding and performing intervention/treatment. sEMG can be useful in the following cases: 1. treatment of hypertonic muscles (gastrocnemius, soleus, hamstrings, adductor) with botulinum toxin 2. personalized therapies 3. selective dorsal rhizotomy 4. decision on surgical acts or rehabilitative interventions that involve bandages or constraints on joints 5. functional electrical stimulation (FES), for instance, for ankle dorsiflexors (TA) stimulation during gait 6. in the most serious cases, functional surgery can be used for elongation of targeted muscles or transpositions of tendons/muscles in order to change their function	very likely neutral/do not know very unlikely
Several factors may limit the widespread usage of sEMG in clinical neurorehabilitation. Based on your experience and knowledge, please score the relevance of the following elements as potential barriers to the clinical use of sEMG: 1. lack of widely accepted evidence that the use of sEMG in neurorehabilitation helps the selection of treatments 2. lack of widely accepted evidence that the use of sEMG improves treatment effectiveness 3. lack of normative data for evaluation of children with CP based on sEMG 4. insufficient education/practice for professionals in neurorehabilitation at refresher courses 5. insufficient or lack of education on sEMG at the university 6. limited relevance of sEMG as a clinical tool (sEMG has more theoretical relevance) 7. high cost of sEMG equipment 8. sEMG data analysis/interpretation is difficult to perform without specific education/training 9. sEMG software/device not easy to use or not friendly enough for clinicians 10. time consuming 11. discomfort for children with CP 12. no multidisciplinary team available 13. clinical aim is to associate symptoms to therapy and not to investigate the pathological mechanisms using sEMG 14. EMG measurements do not improve the outcome of treatment	very relevant neutral/do not know not relevant

### Results of Survey

#### Background and Self-Reported Usage of sEMG by Participants

All clinicians that completed survey questionnaires (7 medical doctors, 5 neuro-developmental disorders therapists and 16 physiotherapists, Figure 4A) were highly involved in pediatric neurorehabilitation though their self-selected level of knowledge and usage of sEMG varied. Not all clinicians reported learning the sEMG technique at the university or refresher courses (Figure 4A middle panel). While some respondents (12/28) reported “good” or “very good” level of knowledge about the use of sEMG (Figure 4A, right panel), nevertheless, most participants do not use sEMG in their clinical practice (Figure 4B, right panel).

**Figure 5:**
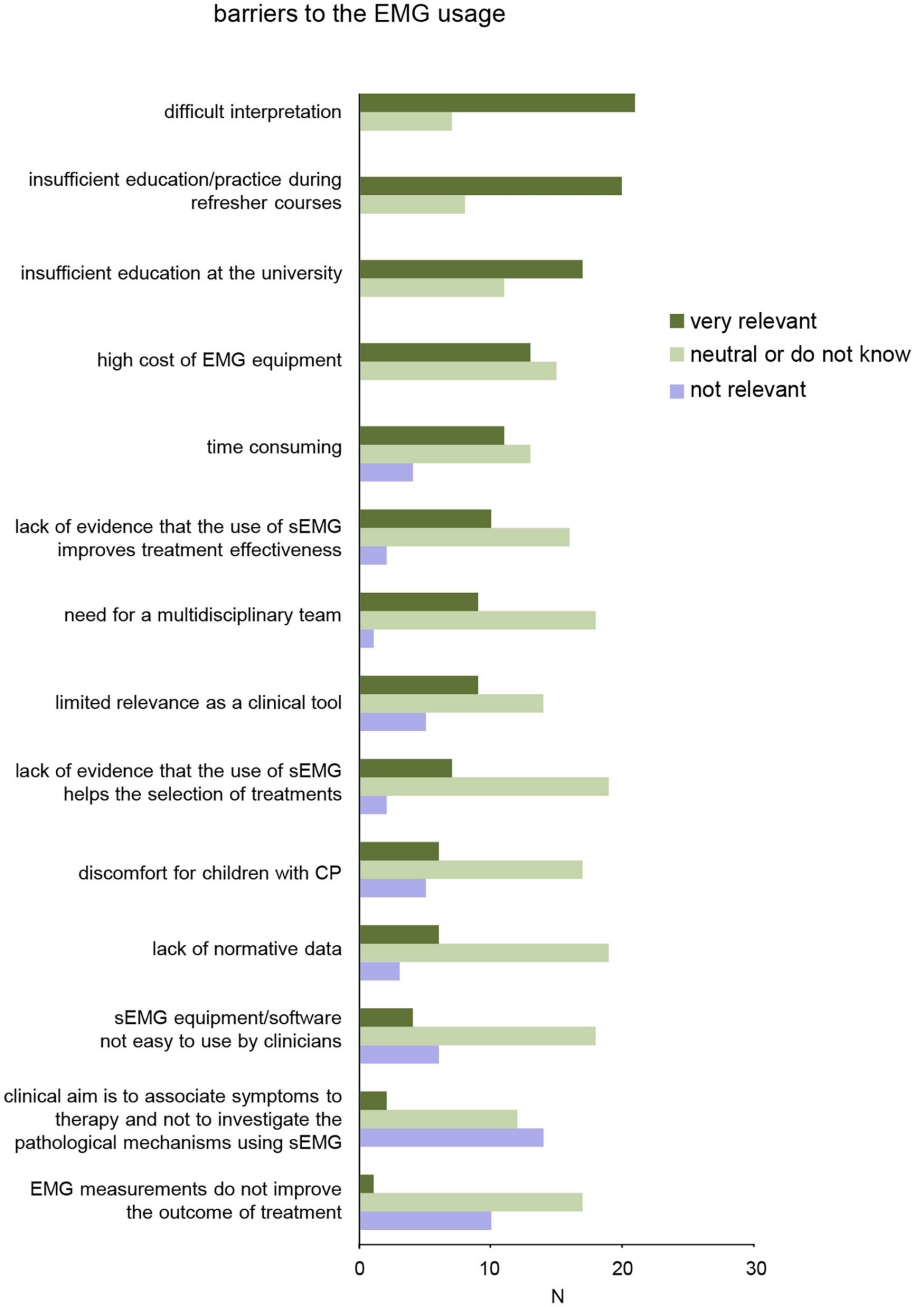
Results of survey for barriers on sEMG use in clinical neurorehabilitation. The items are ordered according to relevance (from top to bottom).

The survey (Table 1) also included two major sets of questions related to the usefulness (Figure 4) and barriers (Figure 5) to the clinical usage of sEMG. We describe the results of the survey below.

#### Usefullness of sEMG

Twelve (43%) contributors totally agree with the statement that sEMG is rarely used in clinical neurorehabilitation (Figure 4B, left panel), the majority of participants agree that sEMG is currently more relevant for researchers than clinicians and also that sEMG provides information on neuromuscular function that is not provided by other assessment techniques/tools in neurorehabilitation (Figure 4B, right panel). While most participants have limited practice with sEMG, they nevertheless expressed willingness to use sEMG to improve their own capacity for neurological assessments (Figure 4B, right panel).

Regarding the role of sEMG in muscle functioning assessment in children with CP, the majority of participants agreed that sEMG may be useful to: outline the abnormal timing of muscular actions during movements (e.g., gait, motor tasks), evaluate muscular fatigue, evaluate the appropriateness of muscle activation in specific motor acts, identify pathological patterns of motor unit behavior, evaluate maximal voluntary activation, characterize involuntary muscle activations (e.g., dystonia, ataxia, spasticity), and characterize muscle fiber conduction velocity (Figure 4C, upper panel). Nevertheless, many of them were also “neutral” or disagreed with these assessments (e.g., for stretch reflex anomalies, etc.).

Regarding the usefulness of sEMG for decision making or performing invasive intervention/treatment, more than half of participants expressed themselves in favor of the sEMG usage in the following circumstances: treatment of hypertonic muscles with botulinum toxin, personalized therapy, selective dorsal rhizotomy, decision on surgical acts or rehabilitative interventions that involve bandages or constraints on joints, FES, functional surgery such as elongation or transpositions of tendons/muscles in order to change or improve their function (Figure 4C, lower panel). On the other hand, about half of them were uncertain or disagreed with these sEMG applications.

In sum, although the participants believe that the application of sEMG in the field of rehabilitation is useful (Figure 4C) and provides information on neuromuscular function not provided by other techniques, many of them were still uncertain about its usefulness (for instance, they consider sEMG to be more relevant for researchers than for clinicians, Figure 4B, middle panel).

#### Barriers to the Clinical Use of sEMG

We also specifically asked the participants about potential barriers to the clinical use of sEMG in neurorehabilitation of children with CP (Table 1, last section). More than 50% of the participants very likely consider the following elements as potential barriers (Figure 5): difficult interpretation of sEMG data without specific education/training (21/28), insufficient education/practice during refresher courses (20/28), and inadequate education and training for physiotherapists and medical doctors on sEMG at the university (17/28). Less than 50% of the participants consider the following elements as potential barriers to the clinical use of sEMG: high cost of sEMG equipment (13/28), time-consuming for sEMG measurements/assessment (11/28), lack of evidence that the use of sEMG improves treatment effectiveness (10/28), limited relevance of sEMG as a clinical tool (9/28), need for a multidisciplinary team (9/28), lack of evidence that the use of sEMG helps the selection of treatments (7/28), lack of normative data for evaluation of impairments in children with CP based on sEMG (6/28), sEMG device/software not easy to use by clinicians (4/28). Nevertheless, only few participants agreed that sEMG measurements should not be used to investigate the pathological mechanisms or do not improve the outcome of treatment (Figure 5, bottom).

## Discussion

In the first section (“Motor impairments and sEMG applications in CP”), we described motor impairments resulting from a lesion occurring in the developing brain and corresponding sEMG applications. The use of sEMG signals through state-of-the-art and advanced methodologies is becoming essential in rehabilitation engineering and in clinical neurophysiology. Many publications in peer-reviewed journals provide various arguments and examples related to the current clinical applications of sEMG showing the information available for neurorehabilitation of children with CP (17, 19, 36, 55, 59, 63, 64, 69, 73, 93–96, 99, 100, 116, 132–136). Recent studies have recommended using a combination of electromyography and biomechanical measurements as a more accurate method of evaluating impaired motor function in individuals with brain damage, such as cerebral palsy (25, 37, 69, 100, 102, 135, 137, 138). The use of sEMG is essential in the decision making of functional surgery and in the assessment of spasticity (133). Moreover, monitoring sEMG signals is beneficial for detecting voluntary muscle activation that may interfere with the identification of reflex responses and evaluation of corticospinal and neuromuscular connectivity (134). The uniqueness of this technology is that it provides information on neuromuscular function not provided by other techniques, assists for intervention or muscle/tendon surgery acts, and evaluates more integrative functioning and impairment of the nervous system based on multi-muscle sEMG recordings. sEMG can be helpful for monitoring neuromuscular modifications and progress in children with CP, integrating the clinical evaluation and providing a picture of both impairment and functional alteration.

Despite these successes and evidences, clinical applications remain very limited because of many barriers acknowledged by the end-users (therapists and medical doctors). In addition, publication of clinical results in the experimental papers or state-of-the-art reviews is necessary but far from being sufficient pointing to the important issue of disseminating rehabilitation innovations and evidence-based practice (139). The results of a survey showed a lack of the sEMG usage in clinical practice and generally limited competence of clinicians in its usage for rehabilitation of children with CP (Figure 4B). A number of barriers limiting the widespread application of sEMG techniques were considered (ranked according to their greatest relevance in Figure 5); among the most relevant – “difficult interpretation” and “insufficient education.”

One limitation of our survey is that the results are for a single center. Nevertheless, the sample of responders was relatively large (*n* = 28) and included experts in pediatric rehabilitation: physical therapists, neuro-developmental disorders therapists, and medical doctors (Figure 4A, left panel). It is also worth noting that many of the barriers for the sEMG use were acknowledged for other populations of patients as well [see, for instance, other articles in this research project: (140–145)]. Therefore, a general need for this innovative technology suggests that “*specific education should be part of the rehabilitation professionals' curriculum*” (142).

Some barriers are related to the lack of confidence or knowledge when comparing the results of sEMG with the diagnostic power of needle EMG (146, 147), or are related to the problem of assessing “function” (with scales and observational descriptions) rather than “impairment” (with measurement of physical quantities) (148, 149). There is often a lack of a common language with rehabilitation engineers and many therapists and medical doctors lack the technical background to interpret the sEMG outcomes. They may believe that time spent in assessing sEMG is not “productive” because it provides limited or incomplete information about pathology. However, this opinion may be related to several reasons including a lack of knowledge of the subject. Some barriers are technical, like difficulties with the application of sEMG, time consuming, signal processing and information extraction algorithms, which do not directly produce clinically relevant information. Among technical problems associated with certain applications of sEMG in children with CP, one could also mention difficulties in normalization of sEMG amplitude to maximum voluntary contraction or distinguishing involuntary stretch reflex activation from voluntary activation. For instance, children with CP demonstrate significantly larger intensity of MN activity of the lumbosacral enlargement during gait than TD children (37). However, it is difficult to evaluate the amount of excessive muscle activation for personalized assessments since differences in “non-normalized” sEMG intensity may reflect potential differences in subcutaneous tissue thickness between subjects. Using reference (rather than maximum) contractions for sEMG normalization may be used in some cases although the order of motor unit recruitment, differences in muscle fiber composition in children, difficulty to activate a particular muscle in isolation and crosstalk from neighbor muscles affect sEMG normalization. One should also keep in mind that it is often difficult to obtain reference muscle contractions in infants at risk of developing motor disorders [e.g., (150–152)]. Finally, the cost of the devices, the reimbursement procedures, and the time needed to perform a measurement and obtain a clinically useful information have also to be taken into account.

These perceived reasons for the potential barriers (Figure 5) do suggest a necessity for additional training sEMG courses and/or need to add specific education in graduate degree courses of physiotherapists and medical doctors. The participants agreed that the sEMG analysis may be difficult to execute without such knowledge and specific training. Moreover, the lack of specific education also prevents the preparation of clinical application guidelines that must become a part of the education of all operators potentially involved in sEMG application. Teachers of physiotherapy and neurology have in general, no or very limited research experience in this area. Insufficient continuing education and involvement of educators in research projects is a barrier to clinical use of all new technologies in general, and sEMG in particular. To overcome technical and education barriers, both better technical competence of clinicians and providing a medical technologist in major hospitals (like adopted in the Netherlands: https://www.tudelft.nl/en/2020/3me/june/clinical-technologists-officially-registered-healthcare-professionals/) may have an impact on increasing the use of sEMG in clinical practice. However, given the primary usage of this information by clinicians, we suggest specific theoretical-practical training to be carried out both during university courses for health rehabilitation professions and during medical specialization courses with outlets in neurorehabilitation. This implies recruitment of specialized professionals as teachers, availability of medical technology in university hospitals and therefore allocation of state or university funds.

## Conclusions

Despite the uniqueness of the sEMG technology and the successes in clinical applications for planning and assessment of treatment of children with cerebral palsy, clinical application and practice in rehabilitation departments remain very limited because of many barriers. Various educational and technical barriers to a widespread use of sEMG were acknowledged by the end-users (therapists and medical doctors). Overcoming these barriers requires a highly interdisciplinary educational approach. Rehabilitators and engineers should have overlapping education and this would lead to overcoming the existing communication gap developing a common language and promoting the use of sEMG systems.

## Author Contributions

All authors listed have made a substantial, direct and intellectual contribution to the work, and approved it for publication.

## Conflict of Interest

The authors declare that the research was conducted in the absence of any commercial or financial relationships that could be construed as a potential conflict of interest.
